# Targeting *PTEN* but not *SOCS3* resists an age-dependent decline in promoting axon sprouting

**DOI:** 10.1016/j.isci.2022.105383

**Published:** 2022-10-17

**Authors:** Cédric G. Geoffroy, Jessica M. Meves, Hugo Jae Mun Kim, Daniel Romaus-Sanjurjo, Theresa C. Sutherland, Jeffrey J. Li, Juliet Suen, Joshua J. Sanchez, Binhai Zheng

**Affiliations:** 1 Department of Neurosciences, School of Medicine, University of California San Diego, La Jolla, CA, USA; 2 Department of Neuroscience and Experimental Therapeutics, College of Medicine, Texas A&M University, Bryan, TX, USA; 3 Neurosciences Graduate Program, University of California San Diego, La Jolla, CA, USA; 4 VA San Diego Research Service, San Diego, CA, USA

**Keywords:** Molecular neuroscience, Cellular neuroscience

## Abstract

Axonal repair is critical for functional recovery after injury of the CNS. We previously reported that neuronal *PTEN* deletion exhibits an age-dependent decline in promoting axon regeneration from the corticospinal tract (CST). How sprouting of uninjured axons, a naturally occurring form of axonal repair, is impacted by age is unknown. We assessed CST sprouting after unilateral pyramidotomy in *PTEN* and/or *SOCS3*-deleted mice at different ages. While *PTEN* deletion enhances sprouting independently of age, *SOCS3* deletion loses its sprouting-promoting effect with age. The synergistic effect of *PTEN*/*SOCS3* co-deletion on CST sprouting is rapidly lost with increased age. Overall, promoting sprouting appears more robust across age than regeneration, yet distinct molecular pathways are differentially impacted by age. Importantly, six-week delayed *PTEN* deletion promotes CST sprouting across age groups, supporting a clinically relevant time frame for this neural repair strategy independently of age.

## Highlights

• Neuronal *PTEN* deletion promotes robust CST axon sprouting in middle-aged mice
• *SOCS3* deletion loses effectiveness in promoting axon sprouting in middle-aged mice
• *PTEN* and *SOCS3* co-deletion rapidly loses synergy with increased age
• Delayed *PTEN* deletion in adult mice promotes CST axon sprouting across age groups

## Graphical abstract

**Figure:**
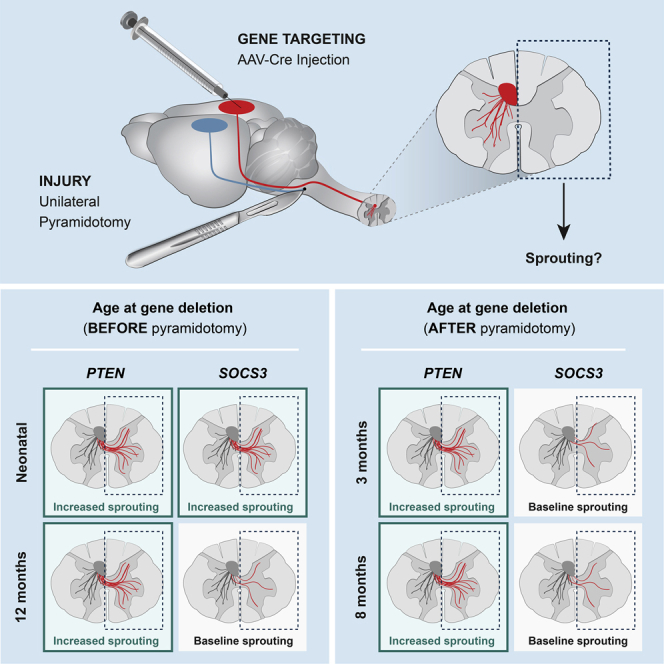

## Introduction

Enhancing axonal repair is a central theme in developing restorative therapies for spinal cord injury as functional deficits and paralysis are primarily caused by axonal damage. Substantial advances have been made in the past decade to understand the molecular regulation of axonal growth after CNS injury (He and Jin, 2016; Sofroniew, 2018). However, a dichotomy exists in age between animal studies and the human populations who suffer from spinal cord injury. While experimental studies predominantly rely on the use of young adult animals, people who live with a paralyzing spinal cord injury are trending significantly older in recent decades. In the United States, the average age at the time of a spinal cord injury has increased from 29 years in the 1970s to 43 in recent years (NSCISC, 2019). The average age of Americans who live with a paralyzing spinal cord injury is now 48, with the predominant age group at 40–59 (representing 55% of the total) corresponding to middle age (CDRF, 2009). Hence, on the one hand, age is widely recognized as an important biological variable by researchers and funding agencies that impacts experimental outcomes in animal models of human conditions. On the other hand, our understanding of the age impact on many biological processes and axonal repair, in particular, is rather limited due to the substantial time and effort required to study older animals, and even more so when complex genetically modified mice are involved.

Using an enhanced regeneration background with neuronal *PTEN* deletion, we previously documented an age-dependent decline in axon regeneration after spinal cord injury (Geoffroy et al., 2016). Neuronal *PTEN* deletion is known to promote corticospinal tract (CST) axon regeneration after spinal cord injury via activating the mTOR pathway (Liu et al., 2010). We found that *PTEN* deletion remains effective in elevating mTOR activity, neuronal soma size, and axonal growth rostral to the injury site in middle-aged mice; however, axon regeneration beyond the injury site was greatly diminished in middle-aged mice (Geoffroy et al., 2016). Corroborating evidence was obtained for both the CST and the rubrospinal tract, with each assessed in a different laboratory, suggesting that this age-dependent decline in regeneration is generally applicable.

Whereas regeneration is axonal growth from injured neurons, sprouting is axonal growth from uninjured neurons often as a compensatory mechanism (Figure S1A) (Geoffroy and Zheng, 2014; Tuszynski and Steward, 2012). Both may contribute to functional recovery. Compared with regeneration, sprouting occurs naturally after CNS injury and can be modulated more readily by molecular intervention (Bradbury and McMahon, 2006; Cafferty et al., 2008; Geoffroy et al., 2015; Geoffroy and Zheng, 2014; Hutson and Di Giovanni, 2019). As such, we hypothesized that the sprouting-enhancing effect of molecular interventions would be more likely to resist an age-dependent decline. We tested this hypothesis by examining CST axon sprouting in *PTEN* and/or *SOCS3*-deleted mice at different ages before, or following a unilateral pyramidotomy injury. Results indicate that *PTEN* but not *SOCS3* deletion resists an age-dependent decline in promoting CST axon sprouting, revealing a pathway-dependent effect of age on sprouting. Furthermore, the sprouting-enhancing effect of *PTEN* deletion can sustain a six-week delay post-injury regardless of age, highlighting the clinical relevance of this strategy for neural repair.

## Results

### The experimental setup

The central question we set out to address is how age impacts the sprouting-enhancing effect of molecular interventions such as *PTEN* deletion. We applied the unilateral pyramidotomy injury to examine CST axon sprouting (Figure S1B). This is a well-established injury model where one side of the CST is severed at the level of medullary pyramids above pyramidal decussation, and sprouting from the spared (uninjured) side across the midline into the gray matter of the denervated side at the cervical level is then examined with axon tracing (Geoffroy et al., 2015; Lee et al., 2010; Liu et al., 2010). We note that in the literature sprouting may also refer to collaterals branching off an injured axon proximal to the injury site (i.e., regenerative sprouting). However, the current study focuses exclusively on axonal sprouting from uninjured neurons. Neuronal *PTEN* deletion is known to promote CST sprouting (Liu et al., 2010). Neuronal *SOCS3* deletion synergizes with *PTEN* deletion in promoting CST sprouting when initiated at the neonatal stage, which represents one of the most potent molecular manipulations in enhancing CST sprouting reported to date (Jin et al., 2015).

To capture the effect of gene deletion on CST sprouting more accurately, we used an inducible tdTomato reporter line to trace CST axons instead of the traditional chemical tracer biotinylated dextran amine (BDA) so that gene deletion and axon tracing would coincide in the same set of neurons. Specifically, we bred *PTEN*^*f/f*^ and/or *SOCS3*^*f/f*^ mice to the *tdT*^*f/f*^ mouse line that carries a Cre-inducible tdTomato reporter at the *ROSA26* locus: *ROSA26-CAG-loxP-STOP-loxP-tdTomato-WPRE* (shortened as *tdT*^*f*^ below) (Madisen et al., 2010). The resulting mice of *PTEN*^*f/f*^*;tdT*^*f/f*^*, SOCS3*^*f/f*^*;tdT*^*f/*f^and *PTEN*^*f/f*^*;SOCS3*^*f/f*^*;tdT*^*f/f*^ genotypes along with non-mutant *tdT*^*f/f*^ controls were injected with AAV-Cre at different ages in the sensorimotor cortex to induce *PTEN* and/or *SOCS3* deletion with concomitant induction of tdTomato expression for axon tracing (Du et al., 2015; Lee et al., 2014). This allowed us to trace axons only from CST neurons that had undergone the intended genetic manipulations, which represented an advantage over BDA-based axon tracing (in addition to saving one brain surgery otherwise required for BDA tracing). To boost signals for the optimal detection of axons and especially fine collaterals, we immunostained tdTomato instead of relying on the native tdTomato signals directly.

For simplicity, we used the following genotype designations for Cre injected mice where appropriate: WT (wild-type, with *tdT*^*f/f*^), *PTEN* KO (*PTEN*^*f/f*^;*tdT*^*f/f*^), *SOCS3* KO (*SOCS3*^*f/f*^;*tdT*^*f/f*^) and *PTEN;SOCS3* KO (*PTEN*^*f/f*^;*SOCS3*^*f/f*^;*tdT*^*f/f*^). To quantify CST sprouting, sprouting axon number indices were calculated by counting the numbers of axons crossing pre-defined vertical lines from the midline on C7 transverse sections up to the lateral edge of the denervated gray matter, which were then normalized against the total number of CST axons labeled in the medulla as previously described (Figure S1B) (Geoffroy et al., 2015). In all experiments, we observed a similar number of axons labeled at the medulla level for the young and middle-aged mice, suggesting that the viral transduction efficiency was not altered by the age factor. In addition, any modest differences in labeling efficiency are accounted for in quantifying axon sprouting by the normalization procedure described above.

### Neuronal *PTEN* remains effective in middle-aged mice to enhance corticospinal tract axon sprouting

**Figure 1:**
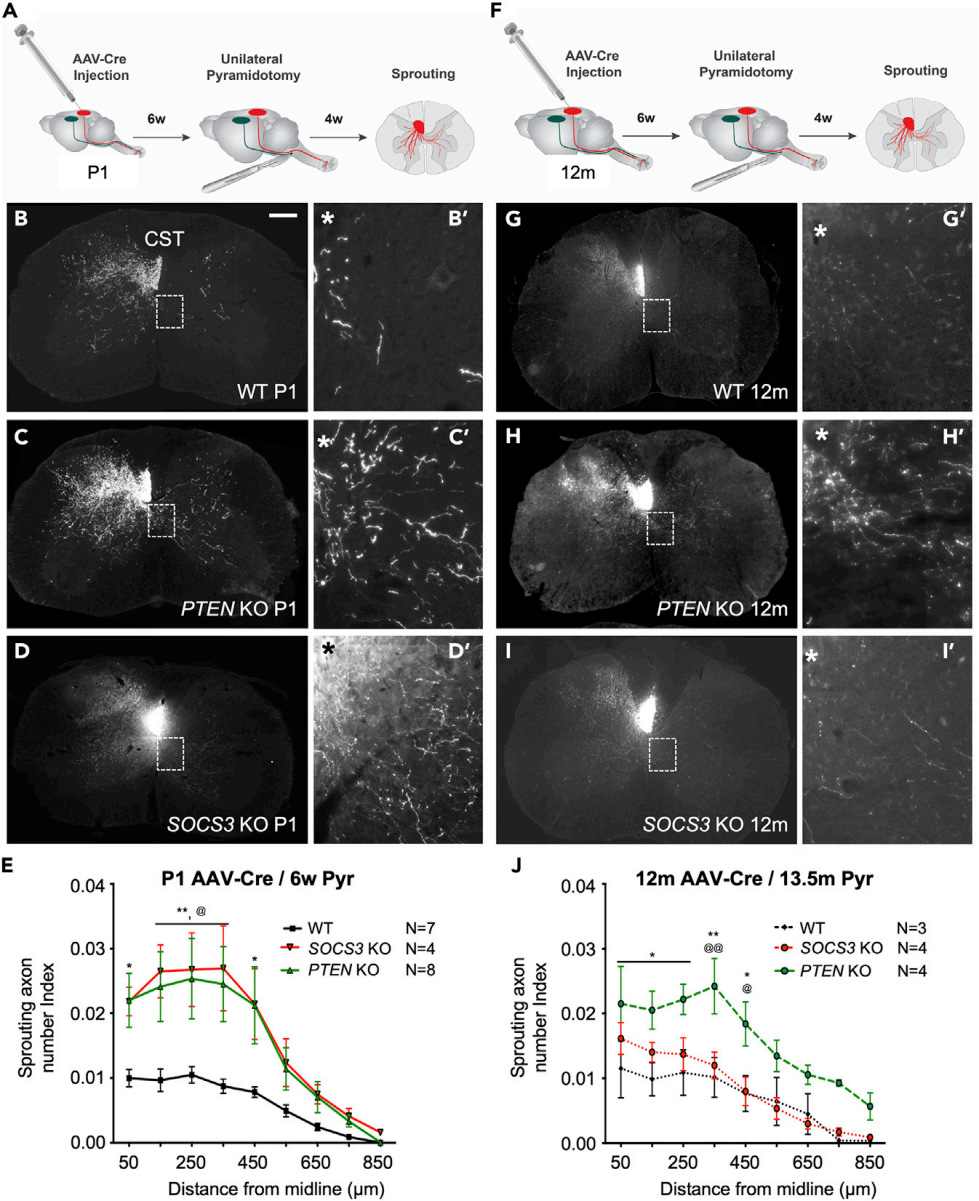
*PTEN* but not *SOCS3* deletion enhances sprouting independently of age (A and F) Schematic representations of the experimental design; gene deletion was induced at P1 (A) or 12 months (F), 6 weeks before pyramidotomy, with AAV-Cre cortical injections. Sprouting was assessed 4 weeks after pyramidotomy. (B–D) Representative images of CST sprouting phenotype at C7 after postnatal day 1 (P1) single gene deletion 6 weeks before pyramidotomy, targeting *PTEN* (C-C′) or *SOCS3* (D-D′); *PTEN* or *SOCS3* deletion significantly enhanced sprouting compared to WT control (B-B′). (E) Sprouting axon number indices on the contralateral, denervated side of the spinal cord expressed as a function of the distance to midline after AAV-Cre injection at P1 and pyramidotomy at 6 weeks of age. There was a significant increase in sprouting in the *PTEN* KO group compared to WT from midline to 450 μm, and in the *SOCS3* KO group compared to WT from 150 to 350 μm. Stats: two-way ANOVA: Tukey’s multiple comparisons test was used to determine the differences between the groups. Data presented as means ± SEM, N = 7 (WT P1), 8 (*PTEN* KO P1), 4 (*SOCS3* KO P1). WT P1 vs. *PTEN* KO P1: ∗p = 0.01–0.05; ∗∗p = 0.001–0.01; WT P1 vs. *SOCS3* KO P1: ^@^p = 0.01–0.05. (G–I) Representative images of CST sprouting phenotype at C7 after middle-age (12-months-old) gene deletion 6 weeks before pyramidotomy, targeting *PTEN* (H-H′) or *SOCS3* (I-I′); *PTEN* deletion but not *SOCS3* deletion, significantly enhanced sprouting. (J) Sprouting axon number indices on the contralateral, denervated side of the spinal cord expressed as a function of the distance to midline after AAV-Cre injection at 12 months and pyramidotomy at 13.5 months of age. There was a significant increase in sprouting in the *PTEN* KO group compared to WT from 50 to 450 μm, and compared to *SOCS3* KO at 350 and 450 μm. Stats: two-way ANOVA: Tukey’s multiple comparisons test was used to determine the differences between the groups. Data presented as means ± SEM, N = 3 (WT 12m), 4 (*PTEN* KO 12m), 4 (*SOCS3* KO 12m). WT 12m vs. *PTEN* KO 12m: ∗p = 0.01–0.05; ∗∗p = 0.001–0.01; *SOCS3* KO 12m vs. *PTEN* KO 12m: ^@^p = 0.01–0.05; ^@@^p = 0.001–0.01.See also Figure S1.

We previously reported an age-dependent decline in the regeneration of injured CST axons beyond a spinal cord injury site in *PTEN*-deleted mice even though *PTEN* deletion in middle-aged mice still increases the expression of phospho-S6 (pS6) and neuronal soma size, both hallmarks of elevated mTOR activity (Geoffroy et al., 2016). To ascertain how age impacts the sprouting of uninjured CST axons, we compared the effect of *PTEN* deletion in new-born mice (with sprouting assessed at a young adult stage) and middle-aged (12 months old) mice on CST sprouting after unilateral pyramidotomy. For the new-born group, we injected AAV-Cre into the right sensorimotor cortex mice at postnatal day 1 (P1) pups, performed injury 6 weeks later at a young adult stage, and assessed CST sprouting 4 weeks post-injury with the tdTomato axon tracer, when the mice were at ∼10 weeks old (Figure 1A). As expected, CST sprouting into the denervated cervical cord, as quantified with sprouting axon number indices, was substantially higher in *PTEN*-deleted mice than WT controls from 50 μm to 450 μm away from the midline on the denervated side (Figures 1B, 1C and 1E). The slightly over 2-fold increase in CST axon sprouting is consistent with previous reports at the same postnatal stage (Geoffroy et al., 2015; Liu et al., 2010), validating the methodology including the use of tdTomato as the axonal tracer. It is important to note that although gene deletion was initiated at the neonatal stage, sprouting was likely induced only after pyramidotomy at 6 weeks of age; thus, enhanced sprouting in *PTEN*-deleted mice for this age group reflected enhanced sprouting in young adult mice, and not neonatal mice.

For the middle-aged group, we induced *PTEN* deletion in 12-month-old mice six weeks before pyramidotomy and assessed CST sprouting four weeks after injury. We and others have previously shown that initiating neuronal *PTEN* deletion pre-injury in young adult mice (4–6 weeks old) promotes CST axon sprouting (Geoffroy et al., 2015; Lee et al., 2014), but to our knowledge, the current study is the first time that the effect of *PTEN* deletion on sprouting is assessed in middle-aged mice. Neuronal *PTEN* deletion in 12-month-old mice increased sprouting by about 2-folds compared with controls. Similar to the new-born group, the sprouting axon number indices were significantly higher in the middle-aged *PTEN*-deleted mice, from 50 μm to 450 μm away from the midline, compared to age-matched WT controls (Figures 1G, 1H, and 1J). Thus, pre-injury *PTEN* deletion in middle-aged mice increased CST axon sprouting at a level similar to what we observed with neonatal *PTEN* deletion (Figures 1E and 1J). These data indicate that, in contrast to regeneration, the sprouting-enhancing effect of *PTEN* deletion on CST axons resists an age-dependent decline.

### Neuronal *SOCS3* deletion does not enhance corticospinal tract sprouting in middle-aged mice

The data above indicate that *PTEN* deletion resists an age-dependent decline in enhancing CST sprouting. To determine whether this observation can be extended to other signaling pathways, we assessed whether neuronal *SOCS3* deletion in middle-aged mice also remains effective in enhancing CST sprouting. Whereas the PTEN/mTOR pathway regulates protein synthesis, the SOCS3/STAT3 pathway regulates retrograde injury signaling and transcription (Lu et al., 2014); hence, the two pathways regulate different aspects of axonal repair. While PTEN negatively regulates mTOR signaling, SOCS3 negatively regulates STAT3 signaling. Just as for the *PTEN* study, we compared the sprouting-enhancing effect of *SOCS3* deletion in very young mice and middle-aged mice with the same experimental paradigm (Figures 1A and 1F).

As expected, initiating *SOCS3* deletion at P1 induced robust CST axon sprouting after unilateral pyramidotomy in young adult mice, consistent with previous studies (Smith et al., 2009); in addition, the level of enhancement in CST sprouting with neonatal *SOCS3* deletion was comparable to that with neonatal *PTEN* deletion (Figures 1D and 1E). Just as for *PTEN* deletion initiated at P1 above, this enhanced sprouting when SOCS3 deletion was initiated at P1 reflects enhanced sprouting in young adult rather than the neonatal age. Unlike *PTEN* deletion, however, *SOCS3* deletion in middle-aged mice exhibited a striking decline in the level of CST axon sprouting compared with neonatal *SOCS3* deletion (Figures 1I and 1J). Compared with middle-aged WT controls, *SOCS3* deleted mice exhibited a trend for more CST sprouting at 50 and 150 μm from the midline, which did not reach statistical significance (Figure 1J). Thus, targeting neuronal SOCS3 in middle-aged mice loses effectiveness in enhancing CST axon sprouting, as compared with targeting SOCS3 earlier in life. Together with the data on *PTEN* deletion, these results indicate a pathway-dependent effect of age on sprouting-enhancing molecular manipulations.

### *PTEN* and *SOCS3* co-deletion loses synergy rapidly with increased age in enhancing corticospinal tract sprouting

**Figure 2:**
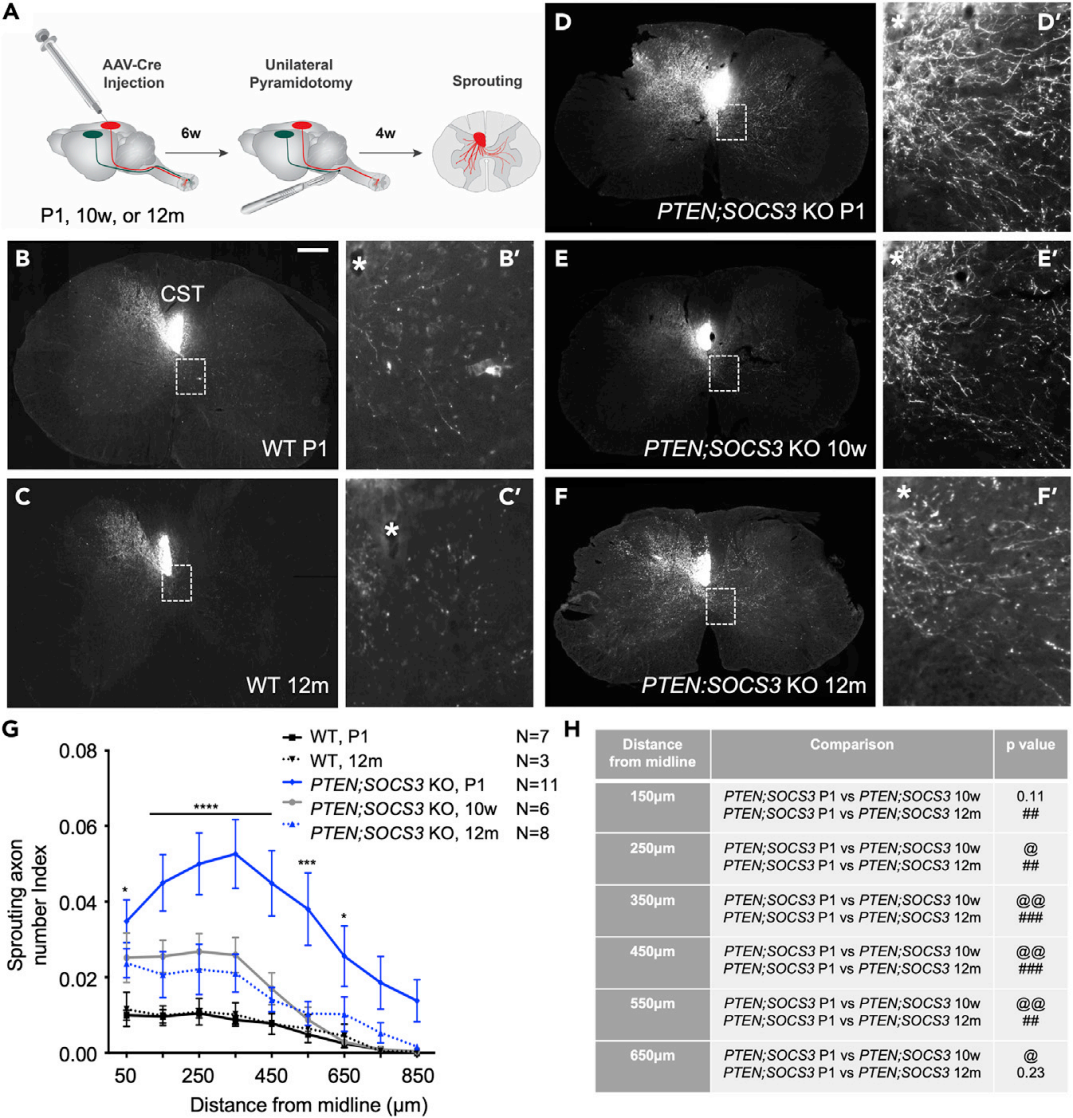
*SOCS3* deletion rapidly loses effectiveness in synergizing with *PTEN* deletion to enhance CST sprouting with increased age (A) Schematic representation of the experimental design; gene deletion was induced with AAV-Cre cortical injections at postnatal day 1 (P1), 10 weeks or 12 months, 6 weeks before pyramidotomy (at 6 weeks, 16 weeks and 13.5 months of age respectively). Sprouting was assessed 4 weeks after pyramidotomy. (B–F) Representative images of CST sprouting phenotype at C7 after *PTEN;SOCS3* co-deletion 6 weeks before pyramidotomy at postnatal day 1 (D-D′), 10 weeks (E-E′) or middle-age (12-months-old, F-F′); *PTEN;SOCS3* co-deletion at P1, but not at 10 weeks or 12 months, synergized with *PTEN* deletion in enhancing CST sprouting. (G) Sprouting axon number indices on the contralateral, denervated side of the spinal cord, expressed as a function of the distance to midline. *PTEN;SOCS3* co-deletion at postnatal day 1 (P1) significantly increased sprouting compared to WT mice from midline to 650 μm; *PTEN;SOCS3* co-deletion significantly increased sprouting compared to *PTEN;SOCS3* co-deletion at 10 weeks and 12 months (significance stated in table H). (H) Table of statistical significance from (G). Stats: two-way ANOVA: Tukey’s multiple comparisons test was used to determine the differences between the groups. Data presented as means ± SEM, N = 7 (WT P1), 3 (WT 12m), 11 (*PTEN;SOCS3* KO P1), 7 (*PTEN;SOCS3* KO 10w), 8 (*PTEN;SOCS3* KO 12m). WT P1 vs. *PTEN;SOCS3* KO P1:∗p = 0.01–0.05; ∗∗p = 0.001–0.01; ∗∗∗p = 0.001–0.01, ∗∗∗∗p < 0.0001; *PTEN;SOCS3* P1 vs. *PTEN;SOCS3* 10w: ^@^p = 0.01–0.05; ^@@@^p = 0.001–0.01; ^@@@^p = 0.001–0.01; *PTEN;SOCS3* P1 vs. *PTEN;SOCS3* 12m: ^#^p = 0.01–0.05; ^##^p = 0.001–0.01; ^###^p = 0.001–0.01. See also Figure S1.

Although either *PTEN* or *SOCS3* deletion induces robust CST axon sprouting in young adult mice when the deletion is initiated at a neonatal stage, only *PTEN* deletion enhances CST sprouting in middle-aged mice. A previous study indicates that neonatal *PTEN* and *SOCS3* deletions synergize to promote a very high level of CST axon sprouting that was accompanied by improved behavioral recovery (Jin et al., 2015). The possibility remained that *PTEN* and *SOCS3* co-deletion in middle-aged mice would also synergize to promote sprouting to levels substantially higher than single *PTEN* deletion, thereby revealing the effect of the additional *SOCS3* deletion. To test this possibility, we examined the sprouting-enhancing effect of initiating *PTEN*;*SOCS3* co-deletion across three age groups: neonates (P1), 10-week-old and 12-month-old mice (Figure 2A).

As expected, initiating neonatal co-deletion of *PTEN* and *SOCS3* increased CST sprouting to a very high level in young adult mice, which was substantially greater than that of either *PTEN* or *SOCS3* single gene deletion (Figures 2D and 2G; Figure 1E). The synergy was especially prominent at distances beyond 500 μm into the denervated gray matter, suggesting that double gene deletion promoted axon sprouting further in distance. Indeed, the sprouting axon number indices in the denervated side significantly increased over 3-folds compared to *PTEN* or *SOC3* single deletion (Figure 2G), consistent with a previous report (Jin et al., 2015). In contrast, for the middle-aged group, CST sprouting was substantially reduced compared with the neonatal *PTEN;SOCS3* co-deletion (Figures 2F and 2G). Indeed, *PTEN;SOCS3* deletion in 12-month-old mice enhanced sprouting to levels comparable to single *PTEN* deletion alone (Figures 2G and 1J), indicating no additional beneficial effect of deleting *SOCS3* at this age.

To explore approximately when this synergy between *PTEN* and *SOCS* deletion is lost, we assessed the effect of initiating *PTEN;SOCS3* co-deletion at 10 weeks of age on CST sprouting as an intermediate time point. To our surprise, there was no significant difference in CST sprouting when *PTEN*;*SOCS3* co-deletion was initiated at 10 weeks versus 12 months of age (Figures 2E–2G). Levels of sprouting in both age groups of *PTEN*;*SOCS3* doubly deleted mice were comparable to single *PTEN* deletion at any age tested (Figures 2E–2G; Figure 1).

Taken together, these data indicate that while *PTEN* deletion resists an age-dependent decline in enhancing CST axon sprouting, *SOCS3* deletion loses effectiveness rapidly with increased age. Furthermore, the synergy between *PTEN* deletion and *SOCS3* deletion is lost by ∼4 months of age.

### Delayed *PTEN* deletion at a chronic time point promotes sprouting across age groups

**Figure 3:**
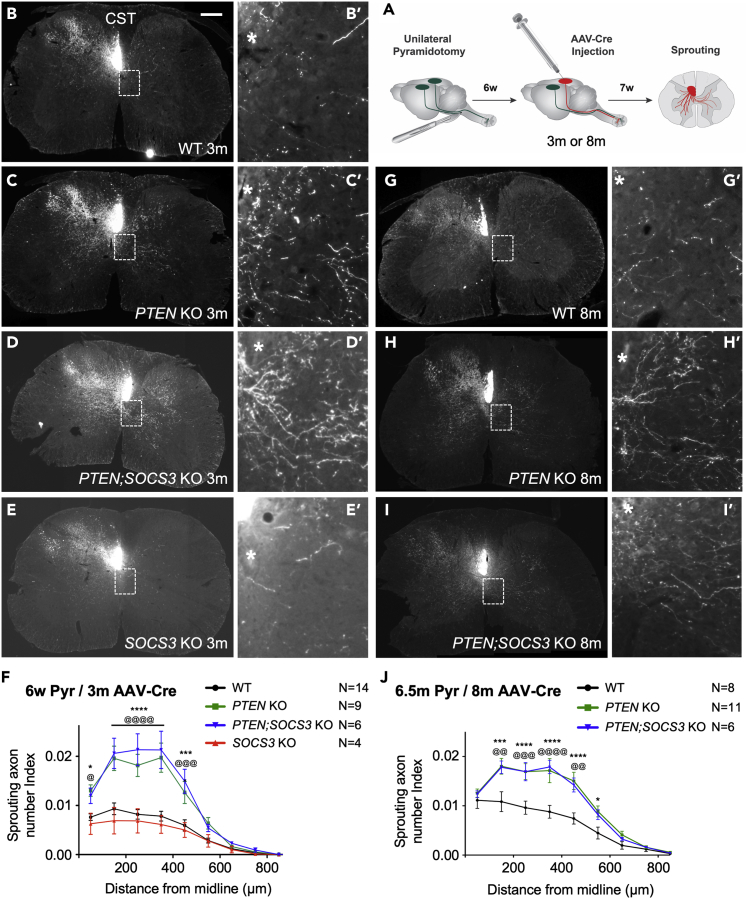
Delayed *PTEN* deletion enhances CST sprouting independently of age (A) Schematic representation of the experimental design; gene deletion was induced at 3 or 8 months, 6 weeks after pyramidotomy in adult mice, with AAV-Cre cortical injections. Sprouting was assessed 7 weeks after cortical injections. (B-E) Representative images of CST sprouting phenotype at C7 after *PTEN* (C-C’), *SOCS3* (E-E′) single deletion or *PTEN;SOCS3* co-deletion (D-D′) in 3-months-old adult, 6 weeks after pyramidotomy; *PTEN* single deletion and *PTEN;SOCS3* co-deletion enhanced CST sprouting to the same level, while *SOCS3* deletion did not appear to have any overt effect compared to WT (B-B′). (F) Sprouting axon number indices on the contralateral, denervated side of the spinal cord expressed as a function of the distance to midline after pyramidotomy at 1.5 months and AAV-Cre injection at 3 months of age. *PTEN* single deletion and *PTEN;SOCS3* co-deletion significantly increased sprouting compared to WT and *SOCS3* deletion from midline to 450 μm. Stats: two-way ANOVA: Tukey’s multiple comparisons test was used to determine the differences between the groups. Data presented as means ± SEM, N (at 3months) = 14 (WT), 9 (*PTEN* KO), 6 (*PTEN;SOCS3* KO), 4 (*SOCS3* KO). WT and *SOCS3* KO vs. *PTEN* KO: ∗p = 0.01–0.05; ∗∗p = 0.001–0.01; ∗∗∗p = 0.001–0.01; ∗∗∗∗p < 0.0001; WT and *SOCS3* KO vs. *PTEN;SOCS3* KO: ^@^p = 0.01–0.05; ^@@^p = 0.001–0.01; ^@@@^p = 0.001–0.01; ^@@@@^p < 0.0001. (G-I) Representative images of CST sprouting phenotype at C7 after *PTEN* (H-H′) or *PTEN;SOCS3* co-deletion (I-I′) in 8-months-old mice, 6 weeks after pyramidotomy; *PTEN* single deletion and *PTEN;SOCS3* co-deletion significantly enhanced CST sprouting compared to WT (G-G′), to similar levels. (J) Sprouting axon number indices on the contralateral, denervated side of the spinal cord expressed as a function of the distance to midline after pyramidotomy at 6.5 months and AAV-Cre injection at 8 months of age. *PTEN* single deletion and *PTEN;SOCS3* co-deletion significantly increased sprouting compared to WT up to 450 μm. Data presented as means ± SEM, N (at 8 months) = 8 (WT), 11 (*PTEN* KO), 6 (*PTEN;SOCS3* KO). WT and *SOCS3* KO vs. *PTEN* KO: ∗p = 0.01–0.05; ∗∗p = 0.001–0.01; ∗∗∗p = 0.001–0.01; ∗∗∗∗p < 0.0001; WT and *SOCS3* KO vs. *PTEN;SOCS3* KO: ^@^p = 0.01–0.05; ^@@^p = 0.001–0.01; ^@@@^p = 0.001–0.01; ^@@@@^p < 0.0001. See also Figure S1.

The above experiments only tested molecular manipulations before injury, which was not a clinically relevant time point. To our knowledge, the most delayed test of *PTEN* deletion on CST sprouting in the literature was a one-week delay (a subacute time point) and it was in young adult mice (Du et al., 2015). To determine whether the effect of *PTEN* deletion on CST sprouting can be extended to clinically relevant time points across age groups, we assessed the sprouting-enhancing effect of *PTEN* deletion 6 weeks post-injury at two different ages. Specifically, we performed unilateral pyramidotomy on mice at either 6 weeks or 6.5 months of age, initiated gene deletion (*PTEN, SOCS3* or *PTEN-SOCS3*) 6 weeks later at 3 and 8 months of age respectively, and assessed CST sprouting 7 additional weeks later (Figure 3A).

Delayed *PTEN* deletion in 3-month-old mice induced CST axon sprouting to a level similar to what was observed when *PTEN* deletion was performed before injury at any of the ages tested (Figures 3B–3F). The sprouting axon number indices of *PTEN*-deleted mice were increased by ∼2-folds compared to 3-month-old WT mice (Figure 3F). In contrast, delayed *SOCS3* deletion at 3 months of age did not increase CST sprouting compared to WT. Delayed *PTEN-SOCS3* co-deletion in 3-month-old mice increased CST axon sprouting at a level similar to the corresponding *PTEN* single deletion at the same age, with ∼2-fold increase compared to WT. Thus, delayed *PTEN* deletion at a relatively young age remains effective in enhancing CST sprouting, but delayed *SOCS3* deletion at the same age loses effectiveness on its own and does not provide any additional effect (synergistic or additive) when co-deleted with *PTEN*.

When gene deletion was initiated at 8 months of age, both delayed single *PTEN* deletion and delayed *PTEN;SOCS3* co-deletion led to a ∼2-fold increase in CST sprouting when compared with WT controls (Figures 3G–3J). Just as the 3-month-old group, *PTEN;SOCS3* co-deletion did not lead to more CST sprouting than single *PTEN* deletion in the 8-month-old group. Altogether, these data demonstrate that delayed *PTEN* deletion, but not *SOCS3*, can enhance CST axon sprouting in both young and middle-aged mice; however, additional *SOCS3* deletion does not further enhance CST axon sprouting in either age group, echoing results from the pre-injury gene deletion experiments above. Thus, targeting the PTEN/mTOR pathway, but not the SOCS3/STAT3 pathway, to enhance axonal sprouting after incomplete spinal cord injury could be effective at a delayed, clinically relevant time point across age groups including middle age.

### SOCS3 deletion elevates STAT3 signaling independently of age

**Figure 4:**
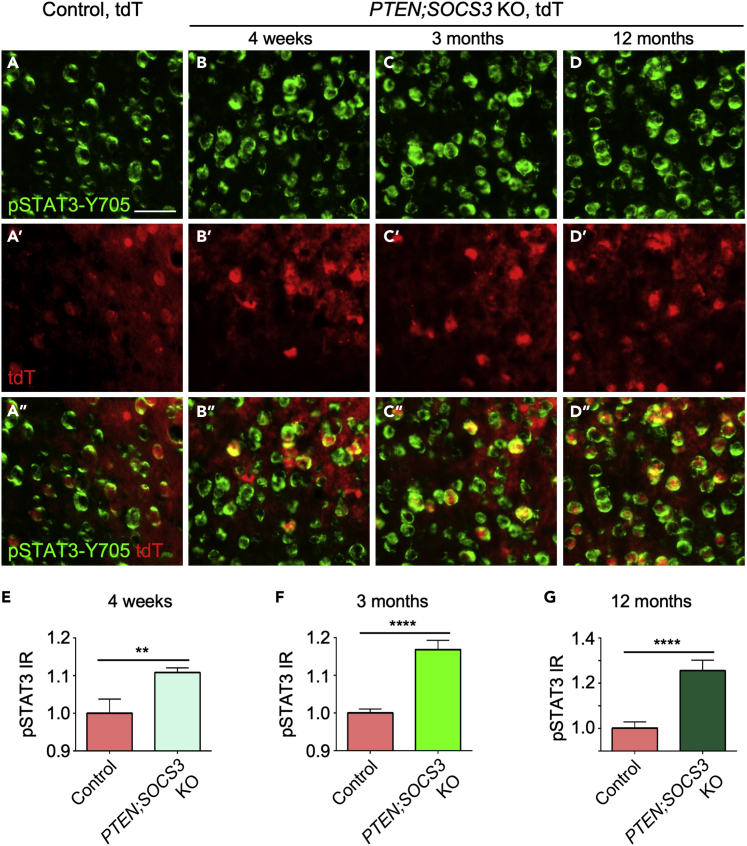
*PTEN;SOCS3* deletion increased STAT3 signaling independently of age (A–D) Representative images of pSTAT3 (at Tyr705) and td-Tomato (tdT) in layer V of the right sensorimotor cortex of 4-week-old (young), 3-month-old (full adult) and 12-month-old (middle-aged) mice (the age at which AAV-Cre was injected, with mice sacrificed 4 weeks later). The control mouse shown was from the 3-month-old group. Scale bars: 50 μm. (E–G) Quantification of the relative pSTAT3-Y705 immunoreactivity (IR) in mice of different genotypes and ages. N = 2 per condition (genotype/age combination). 150 cells were quantified per mouse. Stats: D’Agostino Normality Test; Student’s *t* test (for normally distributed data) or a Mann–Whitney *U* test (for non-normally distributed data). Data presented as means ± SEM Significance level: ∗p = 0.01–0.05; ∗∗p = 0.001–0.01; ∗∗∗p = 0.001–0.01, ∗∗∗∗p < 0.0001). Of note, ANOVA did not show significant differences between the three age groups.

We previously showed that *PTEN* deletion in middle-aged mice remains effective in elevating mTOR signaling (Geoffroy et al., 2016). It remained possible that *SOCS3* deletion becomes less effective in elevating STAT3 signaling with increased age. To test this possibility, we examined pSTAT3-Y705 immunoreactivity in *PTEN*^*f/f*^*;SOCS3*^*f/f*^*;tdT*^*f/f*^ mice following cortical AAV-Cre injection at 4 weeks, 3 months, or 12 months of age. Mice were sacrificed 4 weeks later for immunostaining. Results indicate that *SOCS3* deletion remains effective in elevating pSTAT3-Y705 signaling at increased ages (Figure 4). Thus, a failure to activate the STAT3 pathway through pSTAT3-Y705 is unlikely to underlie the age-dependent decline of CST sprouting in *SOCS3*-deleted mice. Rather, *SOCS3* deletion continues to elevate STAT3 signaling in older mice, but CST sprouting is no longer enhanced through other mechanisms.

## Discussion

In this study, we systemically examined the impact of age on the sprouting-enhancing effects of two molecular manipulations and found that *PTEN* but not *SOCS3* deletion remains effective with increased age. The age-resistant effect of *PTEN* deletion was even observed with delayed gene deletion at six weeks after injury, a time point more conducive for clinical application than acute or subacute time points. Together with our previous report showing an age-dependent decline in CST regeneration in *PTEN*-deleted mice (Geoffroy et al., 2016), the current study indicates that, compared with regeneration, promoting sprouting is likely a more robust neural repair strategy across age. Meanwhile, there is a pathway-dependent effect of age on CST sprouting such that the contribution of each pathway needs to be carefully examined in different age groups to understand the full effect.

Age has been recognized as one of the most important biological variables (along with sex) that broadly impacts experimental outcomes in biomedical research. The dichotomy between the predominantly young animal models and the predominantly older human populations that suffer certain pathological conditions is often cited as a leading contributor to the failure of the clinical translation of promising therapeutic strategies. For instance, age has been proposed as one important factor underlying a large number of unsuccessful clinical trials in the field of stroke (Fisher et al., 2009). Spinal cord injuries used to occur predominantly in younger people, at an average age of ∼29 in the 1970s. This age corresponds to ∼6 months in mice, while 6–10-weeks-old mice are often used as the starting point for regeneration studies. Today, the average age of people who suffer a new spinal cord injury has increased to ∼43 in the US, whereas the average age of people living with a paralyzing spinal cord injury is ∼48, with ∼75% at 40 or older (CDRF, 2009; NSCISC, 2019). Despite its importance, efforts to address the age impact remain limited due to the substantial challenges associated with a protracted experimental timeline—even more so for genetic tests where multiple genetic alleles need to be bred together in the first place. Even for spinal cord injury studies in rats where genetic breeding is typically not required, the average age of animals used is 96 days old and less than 0.35% of the animals are 12 months or older (Nielson et al., 2014) (and Ferguson A., personal communication). This striking dichotomy in age between human spinal cord injury populations and experimental animal models will likely impede the clinical translation of promising restorative therapies.

Müller and colleagues previously reported that geriatric age (≥22 months) reduces the spontaneous sprouting of multiple axonal populations including the CST, serotonergic (5-HT) raphespinal and catecholaminergic (TH) coerulospinal tracts but surprisingly does not diminish the spontaneous regenerative growth of 5-HT, TH and calcitonin gene-related peptide (CGRP)-immunoreactive axons into a spinal cord injury site in rats (Jaerve et al., 2011). Although the exact mechanism remains unclear, transcriptomic analyses revealed substantial differences in axotomy-induced gene expression changes in the sensorimotor cortex between young and old rats (Jaerve et al., 2012). We and others previously showed an age-dependent decline in CST and rubrospinal tract axon regeneration in *PTEN*-deleted mice (Geoffroy et al., 2016). Data from a previous study focusing on chronic injury rather than age suggest that such regeneration can still occur at ≥12 months of age, but at a much slower pace than in younger mice (Du et al., 2015). The current study indicates that *PTEN* deletion enhances CST sprouting at comparable levels regardless of age: from early postnatal to young adult to middle age at 12 months (based on the age when gene deletion is initiated, rather than injury induced or tissue collected). This age-resistant enhancement of CST sprouting by targeting *PTEN* is in stark contrast to its age-sensitive effect on CST regeneration. Therefore, at least when targeting the PTEN/mTOR pathway in CST neurons, enhancing sprouting appears to be a more robust strategy across age than enhancing regeneration. One caveat in comparing our previous regeneration study (Geoffroy et al., 2016) with the current sprouting study is that the former used a thoracic spinal cord injury model while the latter used a pyramidotomy injury in the brainstem. It remains possible that injuries at different locations elicit different levels of axon spouting following the same molecular manipulation, which can be further compounded by age. Future studies are required to exclude this possibility.

Even in the case of regeneration, our previous study indicates that *PTEN* deletion in middle-aged mice remains effective in elevating mTOR activity (as assessed with pS6 immunoreactivity), increasing neuronal soma size and enhancing rostral CST axon growth toward the injury site; yet, regenerating axons could not navigate beyond the injury site as efficiently as in younger mice (Geoffroy et al., 2016). At the time, we suggested extrinsic factors, e.g., glial scar, alteration in inflammation, as a potential cause of this age-dependent decline (Geoffroy et al., 2016). At a superficial level, our current study appears to support that hypothesis because, unlike regenerating axons, sprouting axons do not need to navigate through or around a hostile injury terrain. However, the current data indicate that neuron-intrinsic factors are also at play (Geoffroy et al., 2017), since different signaling pathways (i.e., PTEN/mTOR vs. SOCS3/STAT3) are differentially affected by age in regulating sprouting.

In addition to resisting an age-dependent decline in enhancing CST sprouting when initiated pre-injury, *PTEN* deletion also does so when applied 6 weeks after injury as a model of delayed molecular intervention. Given that most people with spinal cord injury likely have retained some spared axonal pathways (Angeli et al., 2018), targeting *PTEN* to promote sprouting of spared axons could be an attractive strategy for the clinical translation since it is robust across the age and can be applied at a clinically relevant time point. A six-week delay in mice presumably corresponds to even a longer delay in humans, which may allow sufficient time for spinal cord injury patients to prepare physically, mentally, and emotionally for restorative therapies. Sprouting-enhancing interventions would likely need to be combined with other activity-dependent strategies such as rehabilitative training in order to harness functional recovery. In this regard, it is interesting to note that 12 months of age in mice corresponds to about 40 years of age in humans (Dutta and Sengupta, 2016), which is much closer to the prevalent age that is impacted by spinal cord injury in humans than most animal models.

Compared with the age-resistant effect of *PTEN* deletion on CST sprouting, *SOCS3* deletion falls short in enhancing CST sprouting in middle-aged mice, and strikingly this loss may occur as early as ∼10–12 weeks of age when gene deletion was initiated. It should be noted, however, mice in which *SOCS3* deletion was initiated at P1 had pyramidotomy at 6 weeks and then sprouting assessed at 10 weeks of age, thus the enhanced sprouting in these mice reflects the young adult rather than the neonatal age. The synergistic effect of *PTEN* and *SOCS3* co-deletion was lost by 10 weeks of age at the initiation of gene deletion and 16 weeks of age at the time of pyramidotomy. In line with this, initiating *SOCS3* deletion at 3 months of age six weeks *after* pyramidotomy did not enhance CST sprouting. Just as *PTEN* deletion remains effective in elevating mTOR signaling in middle-aged mice (Geoffroy et al., 2016), the current study argues against a simple failure of *SOCS3* deletion to elevate STAT3 signaling (as assessed at Tyr705) as the cause of the age-dependent decline in enhancing CST sprouting. It is possible that *SOCS3* deletion is a weaker manipulation *per se* than *PTEN* deletion in promoting CST sprouting, which is exacerbated by increased age. It is also possible that aspects of STAT3 signaling other than phosphorylation at Tyr705 is affected by age, e.g., phosphorylation at Ser727, or the nuclear entry of STAT3. Additional boosting of this signaling pathway, e.g., by CNTF delivery (Jin et al., 2015; Sun et al., 2011), may be required to enhance sprouting in older mice. Along this line, a previous study indicates that virally mediated overexpression of STAT3 can enhance the sprouting of uninjured CST axons after unilateral pyramidotomy (as well as regenerative CST sprouting rostral to a spinal cord injury although to a lesser extent) in 6–12 week old mice (Lang et al., 2013). How the SOCS3/STAT3 pathway can be manipulated to optimize CST sprouting in adult mice remains to be fully elucidated.

Together, our data indicate that advancing age can negatively impact both the regeneration of injured axons and the sprouting of spared axons after CNS injury, but the impact on regeneration tends to be more pronounced than sprouting. Meanwhile, there is a pathway-dependent effect of age on axonal repair. In particular, targeting the PTEN/mTOR pathway to promote sprouting can be applied at a delayed, clinically relevant time point cross age. Regardless, our data clearly demonstrate the need to test any promising therapies (drug targets, drugs, gene therapies, cell therapies, and so forth) in the age group corresponding to the human population, before any clinical testing. A strategy that promotes axonal growth (regeneration and/or sprouting) and functional recovery in young animals (2–3 months old) may not extrapolate to 6- or 12-month-old animals which correspond to ∼20/25 and 40 years old in humans, respectively. Elucidating the age impact is not only crucial for potential clinical translation, it could also lead to a better understanding of the mechanisms involving different molecular signaling pathways in spinal cord repair.

## STAR★Methods

### Key resources table

**Table:** 

REAGENT or RESOURCE	SOURCE	IDENTIFIER
**Antibodies**		

anti-goat tdTomato	SICGEN	AB8181-200
anti-goat Alexa Fluor 546	Thermo Fisher	A-11056
anti-rabbit pSTAT3-Y705	Cell Signaling	9145
biotinylated anti-rabbit	Vector Laboratories	BA-1000-
anti-mouse PKCγ	Santa Cruz Biotechnology	sc-211

**Bacterial and virus strains**		

AAV-Cre (AAV-2/2)	Salk Institute Viral Vector Core	https://www.salk.edu/science/core-facilities/viral-vector-core/

**Chemicals, peptides, and recombinant proteins**		

Normal Horse Serum	Vector Laboratories	S-2000
Triton X-100	Bio-Rad	1610407
SDS (Sodium n-Dodecyl Sulfate)	Millipore Sigma	428015
Cryo OCT	Fisher scientific	1437365

**Critical commercial assays**		

TSA™ Plus Fluorescein System	Perkin Elmer	NEL741001KT
VECTASTAIN® Elite® ABC-HRP Kit	Vector Laboratories	PK-6100

**Experimental models: Organisms/strains**		

B6.Cg-Gt(ROSA)26Sortm14(CAG-tdTomato)Hze/J	The Jackson Laboratory	007914
PTENf, B6.129S4-Ptentm1Hwu/J,	The Jackson Laboratory	006440
SOCS3f, B6;129S4-Socs3tm1Ayos/J	The Jackson Laboratory	010944

**Oligonucleotides (genotyping primers)**		

ROSA26 Forward: AGGGAGCTGCAGTGGAGTA	The Jackson Laboratory	007914
ROSA26 Reverse: CCGAAAATCTGTGGGAAGTC	The Jackson Laboratory	007914
tdTomato Forward: CTGTTCCTGTACGGCATGG	The Jackson Laboratory	007914
WPRE Reverse: GGCATTAAAGCAGCGTATCC	The Jackson Laboratory	007914
PTEN Forward: CAAGCACTCTGCGAACTGAG	The Jackson Laboratory	006440
PTEN Reverse: AAGTTTTTGAAGGCAAGATGC	The Jackson Laboratory	006440
SOCS3 Forward: CGGGCAGGGGAAGAGACTGT	The Jackson Laboratory	010944
SOCS3 Reverse: GGAGCCAGCGTGGATCTGC	This study	

**Recombinant DNA**		

AAV-CAG-Cre (AAV-2/2)	Zhigang He (Liu et al., 2010).	N/A

**Software and algorithms**		

GraphPad Prism version 9.0	GraphPad software Inc.	https://www.graphpad.com/

**Other**		

2,2,2-Tribromoethanol Avertin 2.5%	Fisher scientific	421430100
Vetbond	3M	50822189
Fluoromount-G	Southern Biotechnology	0100-01

### Resource availability

#### Lead contact

Further information and requests for resources and reagents should be directed to and will be fulfilled by the lead contact, Binhai Zheng (bizheng@health.ucsd.edu).

#### Materials availability

No new materials were generated in this study.

### Experimental model and subject details

#### Mice

Male and female laboratory mice (*Mus Musculus*) aged postnatal day 1–∼14.5 months old were used in all studies. All mice were originally obtained from The Jackson Laboratory: 1) the Rosa26-CAG-loxP-STOP-loxP-tdTomato reporter, Ai14 or B6.Cg-*Gt(ROSA)26Sor*^*tm14(CAG-tdTomato)Hze*^/J, stock # 007914, referred to as *tdT*^*f*^ or tdT (Madisen et al., 2010); 2) the *PTEN* conditional allele *PTEN*^*f*^, B6.129S4-*Pten*^*tm1Hwu*^/J, stock # 006440 (Lesche et al., 2002); 3) the *SOCS3* conditional allele SOCS3^f^, B6; 129S4-*Socs3*^*tm1Ayos*^/J, stock # 010944 (Yasukawa et al., 2003). Mice were backcrossed to C57BL/6J over 9 generations before interbreeding. Mice were interbred to obtain *PTEN*^*f/f*^*;tdT*^*f/f*^*, SOCS3*^*f/f*^*;tdT*^*f/*f^and *PTEN*^*f/f*^*;SOCS3*^*f/f*^*;tdT*^*f/f*^ mice. All the procedures were approved by the Institutional Animals Care and Use Committee at University of California San Diego.

### Method details

#### Viral production and cortical injection for neuronal targeting

As reported previously (Geoffroy et al., 2015, 2016; Meves et al., 2018), AAV-Cre (AAV-2/2) was produced at the Salk Institute Viral Vector Core with HEK293T cells and purified using iodixanol gradients. Detailed viral production and purification protocols can be found at Salk Viral Core’s website. qPCR was used to determine the viral titer; virus was used at 0.5 × 10^12^ GC/mL. Delivery of the virus was performed using modified a 10 μL Hamilton syringe with a fine glass pipette attached to the needle. The syringe was mounted on a stereotaxic device for precise injection. A mix of male and female mice were used for all the groups. For postnatal injections, postnatal day 1 (P1) pups, tdT^f/f^*, PTEN*^*f/f*^*;tdT*^*f/f*^*, SOCS3*^*f/f*^*;tdT*^*f/*^ or *PTEN*^*f/f*^*;SOCS3*^*f/f*^*;tdT*^*f/f*^ mice were cryo-anesthetized, placed on an ice-cold pad and injected with 1 μL of AAV-Cre in the right sensorimotor cortex. After injection, pups were placed on a 37°C warming pad and covered with some home cage bedding to decrease the risk of rejection by the mother. Pups were returned to their mothers once they regained consciousness and normal color and behavior. For adult injections, mice (tdT^f/f^, PTEN^f/f^;tdT^f/f^, SOCS3^f/f^;tdT^f/^ or PTEN^f/f^;SOCS3^f/f^;tdT^f/f^ at different ages) were anesthetized (2.5% Avertin, Sigma) and a right craniotomy performed. A total of 1.2 μL of AAV-Cre (0.4 μL/site) was injected into the right sensorimotor cortex, 0.7 mm deep from the cortical surface, targeting the left forelimb at the following 3 sites: 0.5 mm anterior, 1.2 mm lateral; 0.1 mm anterior, 2.2 mm lateral, and 0.3 mm posterior, 1.2 mm lateral.

#### Corticospinal tract axotomy (pyramidotomy)

Surgeons performing the injury were blind to age and genotype. Unilateral pyramidotomy was performed to assess CST axonal sprouting as previously reported (Geoffroy et al., 2015; Meves et al., 2018). Briefly, mice (*tdT*^*f/f*^, *PTEN*^*f/f*^*;tdT*^*f/f*^, *SOCS3*^*f/f*^*;tdT*^*f/f*^ or *PTEN*^*f/f*^*;SOCS3*^*f/f*^*;tdT*^*f/f*^ at different age) were anesthetized with 2.5% Avertin (Sigma). An incision was made on one side of the trachea to reach the base of the skull and access the medullary pyramids. The entire left pyramidal tract was cut with a feather micro scalpel, just caudal to the foramen magnum. The skin was closed using Vetbond (3M) and mice were placed on soft bedding on a warming blanket held at 37°C until full recovery from anesthesia.

#### Tissue processing

Tissue processing was performed as previously reported (Geoffroy et al., 2015; Meves et al., 2018) and described below. Mice were given lethal dose of Fatal plus, and perfused transcardially with 4% paraformaldehyde. Brains and spinal cords were collected and post fixed overnight at 4°C in the same fixative solution. Tissue was incubated in 30% sucrose for 3 days for cryo-protection. Brain, medulla and different segments of the spinal cord (from C1 to C7) were embedded in OCT compound, and snapped frozen on dry ice. Tissues were sectioned with a cryostat at 20 μm thickness for further processing.

Transverse sections of the medullary pyramids were processed to control for labeling efficiency and for counting tdTomato-labeled CST fibers (see below). Selected transverse sections of cervical spinal cord (C5-7) were immunostained for PKCγ (1:100, Santa Cruz Biotechnology) to examine the completeness of the lesion for each animal; mice with PKCγ staining on the injured side and thus incomplete lesion were excluded from the study, as reported previously (Lee et al., 2010).

For immunohistochemistry, anti-goat tdTomato (1:500, SICGEN, AB8181-200) and anti-rabbit pSTAT3-Y705 (1:100, Cell Signaling, 9145) antibodies were used as previously reported (Chen et al., 2018) and described below. Cervical sections were stained with tdTomato only. Sections were blocked with PBS with 0.4% Triton X-100 and 5 Normal Horse Serum (NHS) for 2 h at room temperature followed by overnight incubation of tdTomato. The next day, sections were washed and incubated with secondary anti-goat Alexa Fluor 546 (Invitrogen) for 1 h at room temperature. After several washes with PBS, sections were cover-slipped with Fluoromount-G (Southern Biotechnology). Brain sections were co-stained with tdTomato and pSTAT3-Y705. Sections were pre-treated with 1% NaOH for 20 min at room temperature, washed with PBS, incubated with 0.3% glycine in PBS for 10 min, rinsed with PBS and finally treated with 0.03% sodium dodecyl sulfate (SDS) in PBS for 10 min. After additional washes, sections were blocked in 5% NHS in 0.2% Triton X-100 in PBS (PBS-TX) for 1 h at room temperature, and then incubated with anti-pSTAT3 antibody for overnight at room temperature. The next day, sections were washed in PBS- TX and then incubated in biotinylated anti-rabbit (1:250, in PBS) for 2 h at room temperature. After several washes, sections were incubated with ABC solution (in 0.1% Tween 20 1X PBS, Vector Laboratories) over night at 4°C. On the third day, sections were washed with PBS (4 times, 30 min each) then with TSA (Alexa Fluor 488, 1:200 in PBS, Perkin Elmer) for 10 min. After several washes in PBS, sections were stained for tdTomato as described above.

#### Microscopy and quantification

Stained tissue sections were photographed using an upright epifluorescence microscopy (Zeiss Axio Imager M1) using the 20X (for sprouting and cortical sections) or 100X (medulla). Sprouting axon number index was quantified using ImageJ as reported previously (Geoffroy et al., 2015; Meves et al., 2018) and described below. Five randomly selected sections between C6/C7 per animal were quantified and averaged. Lines were drawn through the central canal and across the dorsoventral axis, at 50 μm from the midline, then every 100 μm laterally in the denervated side of the gray matter. Numbers of axons crossing theses lines were averaged from five sections and normalized against total axon count in medulla to obtain the sprouting axon number index, which was plotted as a function of the distance from the midline.

Relative pSTAT3-Y705 immunoreactivity (IR) was measured in the tdTomato expressing cortical neurons using ImageJ. At least 150 cells in total were quantified per mouse, from 3 cortical sections. We first performed an internal normalization to the IR from non-tdTomato neurons that surround the tdTomato + neurons, and then this was normalized to the IR from the age-matched controls measured.

### Quantification and statistical analysis

Statistical tests were performed using GraphPad Prism version 9.0. Statistical significance was set at p < 0.05. Post hoc comparisons were carried out only when a main effect showed statistical significance. CST sprouting data were analyzed using a two-way ANOVA followed by Tukey’s multiple comparisons test. Immunostaining intensity data were analyzed using D’Agostino Normality Test followed by a Student’s *t* test (for normally distributed data) or a Mann–Whitney U test (for non-normally distributed data).

## Data Availability

• The data reported in this paper will be shared by the lead contact upon request. • This paper does not report original code. • Any additional information required to reanalyze the data reported in this paper is available from the lead contact upon request.
