# Supplementary material for: Targeting PTEN but not SOCS3 resists an age-dependent decline in promoting axon sprouting
Source: iScience. 2022 Oct 17;25(11):105383. doi: 10.1016/j.isci.2022.105383 (PMC9626739; doi:10.1016/j.isci.2022.105383)
Supplement: Document S1. Figure S1 [file mmc1.pdf]

iScience, Volume *25*

Supplemental information

Targeting *PTEN* but not *SOCS3* resists an age-dependent decline in promoting axon sprouting

Cédric G. Geoffroy, Jessica M. Meves, Hugo Jae Mun Kim, Daniel Romaus-Sanjurjo, Theresa C. Sutherland, Jeffrey J. Li, Juliet Suen, Joshua J. Sanchez, and Binhai Zheng

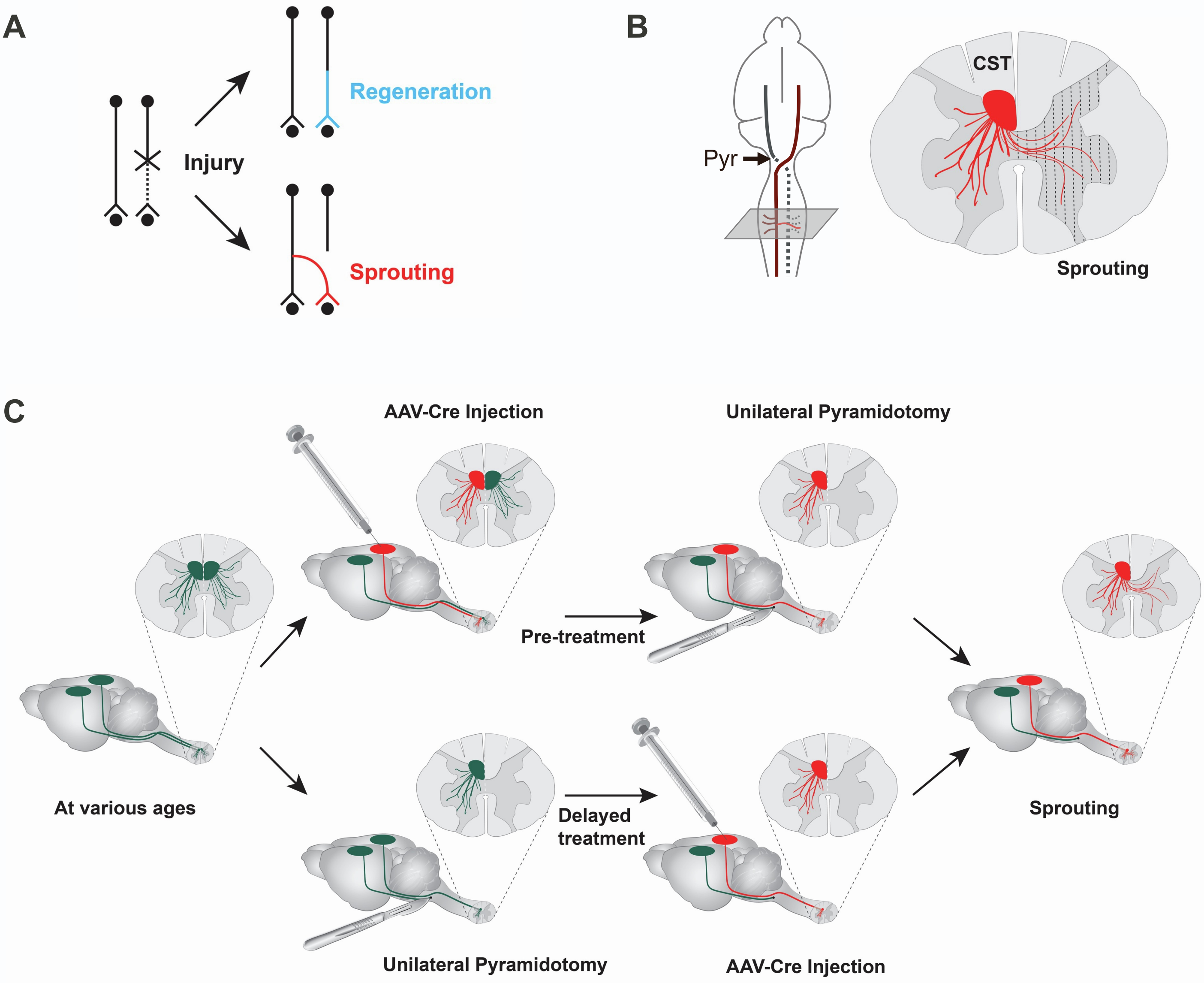

Figure S1. Overview of experimental paradigm to assess CST axon sprouting in this study. Related to Figures 1-3. (A) Illustration of two different forms of neural repair in the CNS after axonal injury: regeneration (blue) is axonal growth from injured neurons; sprouting (red) is axonal growth from uninjured neurons often as a compensatory mechanism. (B) Quantification method for sprouting axon number indices on the contralateral, denervated side of the spinal cord after unilateral pyramidotomy injury. Cre-mediated activation of the tdTomato reporter labels CST axons projecting from the infected corticospinal neurons into the cervical spinal cord (red); lines were drawn through the central canal and across the dorsoventral axis, at 50 µm from the midline, then every 100 µm laterally in the denervated gray matter (vertical dotted line in the right panel). The numbers of tdTomato-expressing axons crossing theses lines were used to obtain the sprouting axon number indices after normalization to the number of tdTomato-expressing axons at the medulla level. (C) Schematic diagram of the experimental flow. Gene deletion was induced with AAV-Cre injections in the right sensorimotor cortex either 6 weeks before pyramidotomy (pretreatment, top panel, at postnatal day 1, 10 weeks or 12 months) or 6 weeks after pyramidotomy (delayed treatment, lower panel, in 3-months or 8-months-old mice). The number of tdTomato expressing sprouting axons was quantified 4 weeks (for pretreatment) or 7 weeks (for delayed treatment) after pyramidotomy.
